# Targeting DNA Damage Response and Repair to Enhance Therapeutic Index in Cisplatin-Based Cancer Treatment

**DOI:** 10.3390/ijms22158199

**Published:** 2021-07-30

**Authors:** Robert Csaba Kiss, Fen Xia, Scarlett Acklin

**Affiliations:** Department of Radiation Oncology, Rockefeller Cancer Institute, University of Arkansas for Medical Sciences, Little Rock, AR 72205, USA; RCKiss@uams.edu (R.C.K.); fxia@uams.edu (F.X.)

**Keywords:** anticancer drugs, cisplatin, DNA damage response, cisplatin resistance

## Abstract

Platinum-based chemotherapies, such as cisplatin, play a large role in cancer treatment. The development of resistance and treatment toxicity creates substantial barriers to disease control, yet. To enhance the therapeutic index of cisplatin-based chemotherapy, it is imperative to circumvent resistance and toxicity while optimizing tumor sensitization. One of the primary mechanisms by which cancer cells develop resistance to cisplatin is through upregulation of DNA repair pathways. In this review, we discuss the DNA damage response in the context of cisplatin-induced DNA damage. We describe the proteins involved in the pathways and their roles in resistance development. Common biomarkers for cisplatin resistance and their utilization to improve patient risk stratification and treatment personalization are addressed. Finally, we discuss some of the current treatments and future strategies to circumvent the development of cisplatin resistance.

## 1. Introduction

Since receiving Food and Drug Administration (FDA) approval in 1978 [1], the widely known platinum-based chemotherapy, cisplatin, has gained clinical indications for the treatment of a broad spectrum of cancers including multiple myeloma, lung, breast, colorectal, gynecologic, and head and neck cancers [1,2]. While cisplatin confers strong cytotoxic activity, especially in combination with other treatment modalities, such as radiotherapy or surgery [3], the development of drug resistance represents one of the major obstacles to the cure of cancer. Even tumors that are initially sensitive to cisplatin may later relapse and develop mechanisms to evade growth restriction and cell death [2]. Cancer control can be further hindered when cisplatin-induced toxicities lead to dose reduction or discontinuation [4].

Resistance to cisplatin has been associated with multiple mechanisms, including alteration of target molecules, production of cisplatin-sequestering proteins, induction of drug efflux pumps, upregulation of DNA repair pathways, and alterations in pro-survival and pro-apoptotic pathways [2,5]. Tumors may also possess multiple mechanisms of resistance simultaneously [5]. Cisplatin exerts its antineoplastic activity primarily through the formation of DNA platination products; therefore, sensitivity of tumors to cisplatin greatly depends on the ability of tumor cells to recognize and repair cisplatin-induced DNA damage. In fact, there is strong evidence to show that the functionality of various DNA repair pathways significantly impacts tumor response to cisplatin treatment [6,7,8,9,10]. Treatment toxicity is similarly mediated by, at least in part, DNA damage and the efficacy of DNA repair pathways in normal tissues.

Circumventing cisplatin resistance and toxicity remains a critical goal in enhancing the therapeutic index of cisplatin and achieving sustained cancer control. Here, we summarize the reported evidence from studies on the mechanism of DNA damage response and repair in cisplatin-mediated cytotoxicity. We additionally discuss the DNA repair pathways and proteins implicated in cisplatin sensitivity and toxicity, as well as predictive markers for cisplatin resistance. These biomarkers might allow for improved risk stratification of patients and selection of more individualized treatment regimens. Finally, we discuss current and future treatment strategies targeting DNA repair pathways that impact therapeutic index of cisplatin treatment of cancers.

## 2. Cisplatin Mechanism of Action in Cancer Control and Toxicity

**Figure 1:**
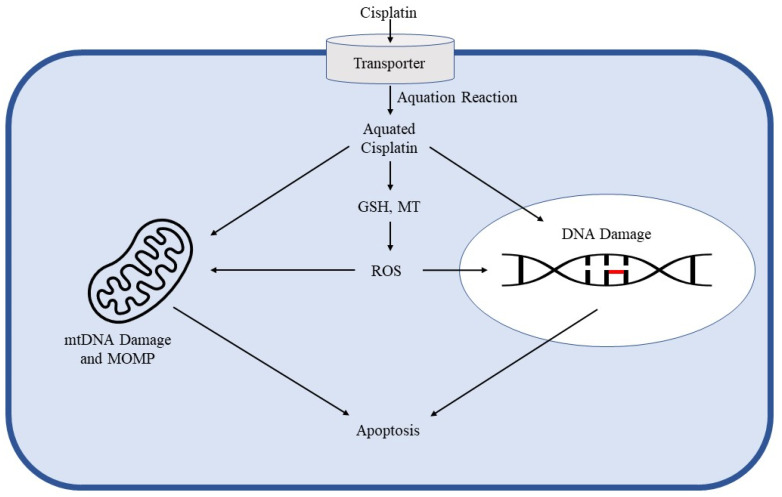
Mechanisms of cisplatin therapeutic action. The dark blue border represents the cell membrane, with the light blue interior being the cytosol and the white oval being the nucleus. Cisplatin first undergoes cellular uptake mediated by copper transporters, indicated by the cylindrical structure crossing the cell membrane. It is then activated in the cytosol through aquation. Aquated cisplatin induces nuclear DNA damage, such as the intrastrand crosslinks represented by the red bar linking two adjacent DNA bases, and mitochondrial DNA damage. The activated drug also attacks reduced glutathione (GSH) and metallothionein (MT) to generate reactive oxygen species (ROS). ROS generation induces apoptosis through mitochondrial outer membrane permeabilization (MOMP) and DNA damage.

Despite decades of research and early identification of DNA as the primary cellular target of cisplatin, the mechanisms involved in cisplatin sensitivity and toxicity remain to be fully elucidated. Cisplatin’s antitumor activity begins with cellular uptake through copper [11], anion [12,13,14], and cation transporters [15,16,17,18]. It is subsequently aquated, allowing it to bind to the guanine N^7^ position on DNA [19]. By doing so, cisplatin generates DNA adducts in a highly conserved manner. The most common of these adducts are 1,2-intrastrand d (GpG) (between adjacent guanine bases on the same DNA strand) and 1,2-intrastrand d (ApG) (between adjacent adenine and guanine bases on the same DNA strand) crosslinks. These adducts distort the helical structure of the DNA molecule, disrupting replication and transcription [20,21,22]. Disruption of nuclear DNA is the most studied mechanism of cisplatin cytotoxicity; however, mitochondrial DNA damage is also indicated. In fact, studies show that cisplatin binds to both nuclear and mitochondrial DNA with the same affinity. Disruption of mitochondrial DNA replication and transcription leads to mitochondrial degradation and apoptosis [2,23]. In addition to DNA damage, cisplatin binds to cytoplasmic molecules, such as glutathione (GSH) and metallothionein (MT). Binding of these substrates results in the generation of reactive oxygen species (ROS) that disrupt mitochondrial membranes, damage DNA, and eventually induce apoptosis [19] (Figure 1).

## 3. DNA Repair Pathways

In response to DNA damage, a DNA damage response (DDR) is activated, resulting in suppression or complete interruption of DNA synthesis, inhibition of the cell cycle, and activation of DNA repair pathways [24]. These pathways are critical to maintaining genome integrity to prevent carcinogenesis [25,26,27,28]. In fact, DDR defects confer increased risk for the development of cancer over a person’s lifetime.

DNA damage is first recognized by molecular “sensors”, most notably ataxia-telangiectasia mutated (ATM), ATM- and Rad3-related (ATR), and DNA-dependent protein kinase (DNA-PK). These proteins are members of the phosphatidylinositol-3-kinase- like (PIKK) family, each regulating DNA repair pathways with different specificities and functions [29,30]. ATM kinase phosphorylates proteins that mediate cell-cycle arrest, DNA repair, and apoptosis in response to recognition of DNA double-strand breaks (DSBs). ATR monitors replication fork progression and responds to a variety of DNA damage that disrupts replication. It activates many proteins to halt the progression of DNA replication and promote DNA repair. DNA-PK is mainly involved in non-homologous end joining (NHEJ) repair and activates a smaller subset of proteins compared to ATM and ATR [5].

The primary DNA repair pathways include homologous recombination (HR), nucleotide excision repair (NER), NHEJ, base excision repair (BER), and mismatch repair (MMR). When cells are defective in one of these pathways, DNA damage accumulates. If the damage is tolerable, the cells will continue to divide, passing erroneous genome to progeny cells. The generation and propagation of an unstable genome is one significant factor contributing to the development of cancer [5]. However, cells that are deficient in one DNA repair pathway are significantly more sensitive to treatment with DNA-damaging agents, including cisplatin. Redundancy does exist among the DNA repair pathways, where tumor cells deficient in one pathway will compensate using other functional pathways. However, cells with a DNA repair pathway defect are generally more sensitive to agents that rely primarily on that pathway [31]. Not surprisingly, numerous proteins involved in the repair pathways have also been implicated in cisplatin resistance. Therefore, a comprehensive understanding of these pathways and their respective proteins is necessary to develop more effective strategies to overcome the challenges of cisplatin resistance and toxicity.

### 3.1. Homologous Recombination

**Figure 2:**
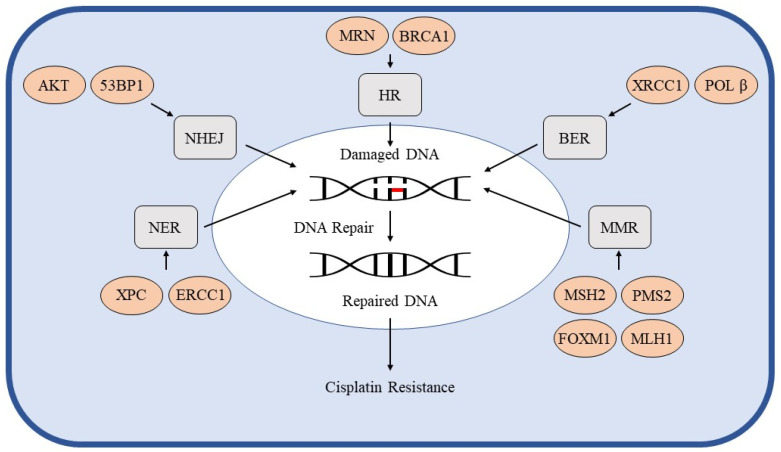
DNA repair pathways and cisplatin resistance. The dark blue border represents the cell membrane, with the light blue interior being the cytosol and the white oval being the nucleus. The grey rectangles represent the mechanisms of DNA damage repair (DDR). The DDR pathways act on damaged DNA to repair the molecule and restore genome integrity. DNA with an intrastrand crosslink is represented in the nucleus by the DNA molecule with a red bar linking two adjacent bases. The repaired DNA lacks the red bar representing the intrastrand crosslink. Each orange oval represents a protein involved in DDR that has been implicated in cisplatin resistance. Arrows point from groups of these DDR proteins to their associated DDR pathway. Enhanced DDR activity in cancer allows for restoration of cisplatin-induced DNA damage and resistance to treatment.

Cisplatin-induced ROS and stalling of the DNA replication fork can produce DNA DSBs that are primarily repaired through one of two mechanisms [32]. HR is the preferred pathway as it is highly precise and preserves the entire DNA sequence. HR involves resection at the broken ends, pairing of the broken DNA strand with a homologous sequence, preferably a sister chromatid, and synthesis of new DNA using the homologous molecule as a template. Activation of cyclin-dependent kinase (CDK), the major cell cycle regulator, induces HR through downstream recruitment of Mre11-Rad50-NBS1 (MRN) complex. MRN complex binds to DNA ends and recruits proteins integral to HR, such as the HR nucleases. ATM is another protein activated by MRN binding to DNA, which activates breast cancer type 1 protein’s (BRCA1) function in cell cycle checkpoint activation [33] as well as cell cycle checkpoint kinase 1 (CHK1). BRCA1 is vital for the ATM-mediated phosphorylation of CHK1 [34]. BRCA1 also indirectly interacts with Rad51, the HR effector protein critical to finding homologous sequences on the sister chromatid [33] (Figure 2).

Many of the proteins within the HR pathway have been implicated in cisplatin sensitivity and resistance. Tumors with somatic mutations in BRCA1 show significantly greater sensitivity to cisplatin treatment [35]. Cells are unable to utilize HR when BRCA1 is absent, resulting in accumulations of DSBs that are lethal to the cell [33]. However, BRCA1-mutated cancers have been found to develop resistance to cisplatin treatment through a secondary mutation that re-establishes BRCA1 function (Figure 2). In fact, tumors possessing this reversion mutation have been shown to develop cisplatin resistance [36]. Besides BRCA1, additional HR proteins have been implicated in resistance development. Overexpression of the MRN complex is associated with cisplatin resistance (Figure 2) while disruption of the complex sensitizes tumors to cisplatin treatment [37,38,39,40,41,42]. Interestingly, a study showed that PD-L1 directly interacts with NBS1 of the MRN complex in cisplatin-resistant head and neck squamous cell carcinomas (HNSCC). Knockdown experiments of either PD-L1 or NBS1 re-sensitized cells to cisplatin treatment, and mutations in both proteins act synergistically to increase cisplatin sensitivity [43]. Moreover, ATM has been implicated in enhanced epithelial-mesenchymal transition and metastatic potential in cisplatin-resistant non-small cell lung cancer (NSCLC) [44], and downregulation of Rad51 has been shown to reduce cisplatin resistance in breast cancer cells [45].

### 3.2. Non-Homologous End Joining

In addition to HR, DSBs can be repaired through NHEJ, a highly efficient repair pathway that is more error prone compared to HR repair. NHEJ has the advantage of being operational throughout the cell cycle, whereas HR is only active during the S/G_2_ phases [46,47,48]. In fact, in G_1_, NHEJ is the dominate repair pathway due to p53-binding protein 1 (53BP1) activity and resulting antagonization of BRCA1. When DNA damage is detected, Ku70/KU80 bind to the exposed breakpoints to recruit nucleases. Detection of DNA damage results in the autophosphorylation of DNA-PK, providing the initial activation of the NHEJ pathway [49]. Phosphorylated DNA-PK is recruited to the DSB site to expose the DNA ends for the nucleases. Because most DSBs do not result in blunt ends, the overhanging single-stranded DNA (ssDNA) must be trimmed by nucleases before X family DNA polymerases (POL) extend the strands. Finally, the ends are ligated using ligase (LIG) 4 bound to XRCC4 [33] (Figure 2).

Although NHEJ is error-prone, it plays a significant role in reestablishing a functional genome following DNA damage. For example, cells lacking XRCC4 have an enhanced sensitivity to DNA damaging chemotherapeutics, including cisplatin [50]. Studies have also shown that cells lacking NHEJ proteins are significantly more sensitive to IR-induced DSBs [46,47,49,51]. Additionally, NHEJ is predominantly active in proliferating cells as opposed to non-proliferating cells. Investigation between the differences in NHEJ functionality in various tumors and healthy differentiated cells can provide useful therapeutic methods that can limit toxicity to healthy tissues [33]. On the other hand, many of the proteins involved in NHEJ are implicated in cisplatin resistance. One study has shown that cisplatin treatment can result in acquired resistance through an AKT-dependent, pro-survival, DNA damage response. This involves AKT translocating to the nucleus and becoming phosphorylated by DNA-PK, resulting in the inhibition of cisplatin-mediated apoptosis [52] (Figure 2). A study has shown that the upregulation of 53BP1 in SKO3 ovarian carcinoma cells resulted in increased resistance to cisplatin [53] (Figure 2). Finally, higher Ku70 expression is associated with a better response to cisplatin treatment and improved overall survival (OS) [54].

### 3.3. Nucleotide Excision Repair

NER is the most common DNA repair pathway involved in the resolution of cisplatin-induced intrastrand DNA crosslinks. Inactivation of this pathway in tumors has shown greater cisplatin sensitivity, OS, and progression-free survival (PFS) [5]. There are two sub-pathways of NER that are intricately coordinated and differ based on the proteins that detect DNA damage. Global-genome NER (GG-NER) utilizes Xeroderma pigmentosum group C (XPC), most significantly XPC-RAD23B, and DNA damage binding complexes that recognize helical distortions from DNA adducts. On the other hand, transcription-coupled NER (TC-NER) is activated when DNA adducts cause RNA polymerase to stall. This activates Cockayne syndrome A and B (CSA and CSB), which bind to and resolve DNA lesions. Both GG-NER and TC-NER require Xeroderma pigmentosum group A (XPA) to interact with ssDNA alterations and recruit TFIIH complex for damage verification [55]. The pathways converge once the TFIIH complex has been recruited to the damaged DNA. The XPB and XPD components of the complex unwind the DNA, creating a 20–30 nucleotide bubble. This is followed by the recruitment of XPA, replication protein A (RPA), and XPG. XPA binds to the 5′ end of the bubble and interacts with other components of NER, including ERCC1-XPF. RPA binds the ssDNA opposite of the lesion to protect it from degradation and helps coordinate the excision and repair processes while XPG provides structural support. Its endonuclease activity is activated only after ERCC1-XPF has been recruited by XPA to the 5′ end. ERCC1-XPF and XPG make incisions at the 5′ and 3′ ends of the damaged ssDNA respectively. POL δ/ε/κ-PCNA-RFC-RPA synthesizes new DNA in replicating (POL ε) and non-replicating cells (POL δ and κ) [56]. The process is completed through strand ligation by LIG1 in replicating cells and LIG3α-XRCC1 in non-replicating cells (Figure 2).

One of the many toxicities involving cisplatin treatment includes chemotherapy-induced peripheral neuropathy (CIPN). This potentially permanent complication is primarily conferred through accumulation of DNA adducts and death of dorsal root ganglia (DRG) neurons [57]. However, many proteins within the NER pathway are involved in the modulation of CIPN, such as apurinic/apyrimidinic endonuclease 1 (APE1), DNA polymerase kappa, XPA, poly (ADP-ribose) polymerase 1 (PARP-1), and sirtuin 2 (SIRT2) [55]. SIRT2 was recently identified as a mediator of NER-dependent neuronal protection against CIPN. Because NER is one of the primary mechanisms for intrastrand crosslink repair, targeting mediators of this pathway could allow for treatment and prevention of cisplatin toxicity [55].

Additionally, many of the proteins involved in NER are associated with cisplatin resistance and sensitivity. These implicated proteins are within both GG-NER and TC-NER. XPC is important in DNA damage recognition by associating with RAD23B to bind cisplatin-induced intrastrand crosslinks and prevent proteasomal degradation. Binding of XPC to DNA is necessary for the recruitment of TFIIH, making it the rate-limiting step of GG-NER [58]. Studies have shown that enhanced expression of XPC increases resistance in many types of cancer [59,60,61]. Additionally, studies involving many different cancer cell lines have shown that the overexpression of XPC leads to cisplatin resistance (Figure 2) while knockout of the gene enhances sensitivity. However, the relationship between XPC upregulation and resistance in cisplatin-treated tumors has not been thoroughly investigated [58]. Within TFIIH are two helicases, XPB and XPD, which are vital for NER function. XPD mutations are commonly found in many human cancers, such as bladder cancer, which are sensitive to cisplatin treatment [58]. XPA is a scaffold protein involved in both GG-NER and TC-NER that is essential to proper assembly of the pre-incision complex, placing endonucleases in the right position for repair of damaged DNA. Metastatic testicular tumors with lower expression of XPA are especially sensitive to cisplatin treatment [62]. ERCC1 is an important protein for recognizing interactions between DNA and XPA and recruiting XPF for heterodimerization. The overexpression of ERCC1 is associated with poor response to cisplatin treatment while underexpression is associated with successful cancer control [58] (Figure 1). Underexpression of ERCC1 has been observed in cells with hypermethylation of the promoter sequence of the ERCC1 gene, but the prevalence of this methylation pattern is unknown [58,63]. Knockdown studies have also shown that depletion of both ERCC1 and XPF disrupts DNA repair from cisplatin treatment and enhances cytotoxicity [64]. Finally, XPG is an endonuclease recruited by TFIIH. Ovarian tumors expressing low levels of XPG have been found to be significantly more responsive to cisplatin treatment. A significant fraction of these tumors displayed methylation within the XPG gene promotor region, but its association with cisplatin response has not yet been studied [58,65].

### 3.4. Base Excision Repair

BER repairs DNA damage not recognized through structural distortions in helical structure. It is active during the G_1_ cell cycle phase and utilizes DNA glycosylases to recognize and remove mutated bases from the DNA molecule. The glycosylases can be monofunctional (specific for a specific nucleotide base) or bifunctional (consisting of glycosylase and β-lyase activity). There are two forms of BER: the short-patch-repair pathway involving monofunctional glycosylases and the long-patch-repair pathway involving bifunctional glycosylases. In the short-patch-repair pathway, a monospecific glycosylase creates an abasic site (site of DNA lacking a nucleotide base). This site is a substrate for the endonuclease APE1, which cleaves directly 5′ and 3′ of the abasic site. POL β then fills the gap with a single nucleotide, and LIG1 or LIG3-XCRR1 ligates the ends together. In the long-patch-repair pathway, bifunctional glycosylases excise faulty nucleotide bases with APE1 carrying out 3′ phosphodiesterase activity. POL 1 (in non-proliferating cells) or POL δ/ε (in proliferating cells) synthesizes the corrected nucleotide sequence, displacing the incorrect strand. The mutated sequence is then removed by the flap endonuclease, and the corrected DNA molecule is ligated via LIG1 [66] (Figure 2).

The components of BER have significant implications on cisplatin treatment. Studies have shown a relationship between cisplatin treatment and the development of idiopathic pulmonary fibrosis (IPF), a condition characterized by accumulation of apoptosis-resistant myofibroblasts. Investigation into this relationship showed that IPF cells treated with cisplatin had heightened activity of XRCC1 through the hyperactivation of CK2. This suggests that cisplatin can worsen pulmonary fibrosis in cancer patients [67]. In addition, cytoplasmic APE1 has been shown to promote cisplatin resistance in osteosarcoma and lung cancer cells [68,69] (Figure 2). Similarly, POLβ P242R germline mutation has been associated with poor response to cisplatin treatment in patients with lung cancer [70] (Figure 2). Taken together, the BER pathway plays a significant role in cisplatin resistance and toxicity.

### 3.5. Mismatch Repair

MMR serves to correct erroneous base insertions from DNA replication and insertion-deletion loops (IDLs) that occur during strand-slippage events, drastically improving the accuracy of DNA replication. MutSα heterodimer (MSH2/MSH6) recognizes mismatched bases and smaller IDLs while MutSβ heterodimer (MSH2/MSH3) recognizes larger IDLs. MutL homologs, particularly the MutLα heterodimer (MLH1/PMS2), are recruited upon identification of a mutation. MutLα has 3’ endonuclease activity, allowing for exonuclease 1 (EXO1) to begin 3’ nick-directed degradation. EXO1 also performs 5’ excision to create a gap in the DNA molecule. This gap is stabilized by RPA. POL δ, replication factor C (RFC), and high mobility group box 1 protein (HMGB1), along with LIG1, synthesize and ligate the corrected DNA strand [66] (Figure 2).

Several associations have been identified between MMR and carcinogenesis. Germline mutations involving components of MMR are linked to the development of Lynch syndrome, also known as hereditary nonpolyposis colorectal cancer (HNPCC). This condition significantly increases the risk of colorectal and endometrial cancers as well as multiple other malignancies [71,72]. Furthermore, an association between MMR function and cisplatin response has been established. A study assessing the relationship between MSH2 and PMS2 expression and cisplatin sensitivity showed that loss of either protein resulted in the development of low-level resistance [73] (Figure 2). In addition, in vitro data has shown MSH3 deficiency sensitizes human colon cancer cells to cisplatin treatment [74]. On the other hand, overexpression of MLH1 leads to enhanced cisplatin sensitivity while the opposite effect is found with underexpression [75] (Figure 2). One study also found that the oncogenic transcription factor FOXM1 is upregulated in cisplatin-resistant ovarian cancer, resulting in direct transcriptional enhancement of the EXO1 gene [76] (Figure 2).

## 4. Predictive Markers of Cisplatin Resistance

Following completion of the International Human Genome Project, attempts to personalize medicine using genomic information have taken off with extraordinary enthusiasm. The concept of precision oncology attempts to select cancer treatment for patients by utilizing diverse techniques to choose more targeted therapies. The use of biomarker testing can help predict prognosis or even direct treatment selection based on variations in single genes or proteins, or a genomic pattern [77]. As a result, the use of molecular predictive markers and genomic profiling have improved the ability to predict whether patients will benefit from various chemotherapies, including cisplatin [78].

### 4.1. ERCC1

ERCC1 is one of the key regulatory proteins within the NER pathway that plays a significant role in the repair of DNA adducts and intrastrand crosslinks (ICLs) generated by cisplatin [79]. ERCC1 overexpression is found in many different types of cisplatin-resistant cancers, such as HNSCC, bladder, and lung cancers [19,80,81]. This makes ERCC1 an important predictive marker for cisplatin treatment. A small study found that half of HNSCC patients with high-ERCC1 expression displayed a poor response to cisplatin treatment leading to significantly lower OS (40%) [82]. A single-center study found that patients with lung adenocarcinoma with high ERCC1 expression had higher tumor, nodes, and metastases (TNM) staging and higher relative risk of death. The study not only showed that low ERCC1 expression was associated with longer OS in all TNM stages, but it also correlated to significantly better response to platinum-based treatment given definitively or in an adjuvant setting [83].

### 4.2. MDR1, MRP1, and β-Catenin

Multidrug resistance protein 1 (MDR1), multidrug resistance-associated protein 1 (MRP1), and β-catenin are proteins involved in drug efflux, a mechanism of resistance that maintains cytosolic concentrations of chemotherapy agents below therapeutic levels. MDR1 and MRP1 are ATP-binding cassette proteins that actively pump drug out of the cell and are regulated by β-catenin [19]. A study found that patients with HNSCC with high expression of MDR1 and MRP1 had significantly lower OS and worse response to cisplatin treatment [84]. A knockdown study of β-catenin demonstrated enhanced cisplatin sensitivity, illustrating a relationship between drug transporters, their regulatory signaling pathways, and cisplatin resistance and sensitivity [19,85].

### 4.3. c-IAP1, XIAP, Apollon, and Livin

Many proteins involved in the inhibition of apoptosis are predictive markers for cisplatin-resistant tumors. Elevated expression of cellular inhibitor of apoptosis protein 1 (c-IAP1) is associated with lymph node metastasis, advanced disease stage, and poor prognosis [86]. 21% of advanced stage HNSCC tissue showed high levels of X-linked inhibitor of apoptosis (XIAP). This correlated to increased cisplatin resistance and poor clinical outcome [87]. Elevated apollon protein and mRNA expression was also found in HNSCC patients who had lower OS and cisplatin resistance [19,88]. It was also shown that knockdown of livin enhances the apoptotic response in HNSCC cells following cisplatin treatment [89]. Taken together, this evidence indicates apoptotic regulatory proteins play a key role in resistance to cisplatin and might be able to predict whether treatment will be successful.

### 4.4. EGFR/FAK/NF-kB Activation

The epidermal growth factor receptor (EGFR)/focal adhesion kinase (FAK)/nuclear factor (NF) -kB pathways are mechanisms by which tumor cells promote proliferation, inhibit apoptosis, and induce drug efflux protein expression. BST2, also known as Tetherin, is one protein that inhibits apoptosis through the NF-kB pathway [19]. It is a strong indicator of cisplatin resistance and poor prognosis based on a study of 117 patients with locally advanced nasopharyngeal carcinoma [90]. KRAS is another protein involved in the EGFR pathway [19]. A study showed that recurrent and/or metastatic HNSCC patients with a specific KRAS-variant (rs61764370, KRAS-variant: TG/GG) experienced poor PFS and marked cisplatin resistance [91].

### 4.5. BRCA1

BRCA1 is a human tumor suppressor protein involved in chromatin remodeling, transcription regulation, protein ubiquitination, cell cycle checkpoint control, apoptosis, and maintenance of genome integrity during the cellular response to DNA damage. Due to its involvement in these functions, defects in BRCA1 are associated with impaired DSB repair and carcinogenesis, particularly breast and ovarian cancer [92,93]. The lifetime risk of breast cancer development in BRCA1- and BRCA2-muation carriers is 45–80%. BRCA1-carriers have a 45–60% chance of developing ovarian cancer. Therefore, BRCA1 genetic testing has significantly improved the ability to risk stratify patients with strong family history and allow them to pursue risk reduction strategies [93]. Because DSB repair pathways are impaired with BRCA1 mutation, BRCA1-mutant cancers are significantly more sensitive to cisplatin treatment. This allows BRCA1 to be a marker for positive response to cisplatin treatment. Unfortunately, it is well established that BRCA1-mutated cancers can develop cisplatin resistance through a secondary mutation that restores BRCA1 function [36,93]. Therefore, simply identifying a patient with BRCA1-mutation does not necessarily indicate positive response to cisplatin treatment, nor does it indicate that the patient will not develop resistance during treatment.

### 4.6. Genomic Analysis

While mutations or alterations in a single gene can aid in prediction of prognosis and treatment resistance, genomic information can provide enough information to guide treatment choices. One of the best characterized clinical applications of genomic sequencing is in early-stage invasive breast cancer, which utilizes a 21-gene panel to assess risk of distant recurrence, chemotherapy benefit, and safety of pursuing only hormonal therapy [94,95,96,97,98,99,100]. A phase III clinical trial showed that a mid-range score on the assay can effectively identify certain patients with early-stage breast cancer that receive no benefit from chemotherapy in terms of disease-free survival when added to adjuvant hormone therapy [101]. Another study used a molecular-based approach to assess whether genomic signature can be used to identify breast cancer patients that can safely be omitted from adjuvant radiation therapy following breast-conserving surgery (BCS) [102].

Genomic profiling continues to show promise as an aid in diagnosis, prognosis, and guidance for treatment in a variety of cancers. These practices can be further utilized to enhance precision medicine and tailor treatments more specifically to the patient’s needs [103,104,105]. One phase II clinical trial was conducted to assess whether a genomic predictor for platinum sensitivity can guide treatment decisions in stage IIIB/IV NSCLC patients. The cisplatin resistance predictive model was developed through analysis of gene expression data in conjunction with treatment response data. Advanced NSCLC patients in the study were assigned to chemotherapy based on the genomic predictor for platinum sensitivity. Squamous cell NSCLCs predicted to be sensitive to cisplatin were assigned to cisplatin/gemcitabine treatment while those identified as resistant were assigned to docetaxel/gemcitabine treatment. Patients with non-squamous NSCLC with predicted sensitivity were assigned to cisplatin/pemetrexed treatment while those found resistant were assigned to pemetrexed/docetaxel treatment. To validate the genomic-based prediction model, the trial evaluated one-year PFS of both cisplatin-sensitive and -resistant groups. Overall time to progression, quality of life, and evaluation of drug sensitivity patterns of cisplatin and pemetrexed were also measured (ClinicalTrials.gov Identifier: NCT00509366). However, the genomics-based predictive model was found to be irreproducible, creating accuracy issues regarding patient assignment to the two cisplatin cohorts and resulting in the termination of the trial. Regardless, the concept of using genomic analysis to guide treatment remains of interest and could have significant impacts on cancer care if these challenges were overcome. It is reasonable to believe that the use of genomic analysis will continue to expand and will eventually help direct treatment decisions when considering cisplatin.

## 5. Current Treatment Strategies and Future Perspectives

Components within each DNA repair pathway show a strong relationship with the resolution of cisplatin-related DNA damage and could serve as targets for overcoming the barriers to cisplatin treatment. One particularly promising approach is the use of small molecule inhibitors to manipulate the DDR and overcome cisplatin resistance [58].

Rapidly dividing cancer cells greatly depend on DSB repair for survival. Resistance to cisplatin and radiation often develops due to augmentation of the DSB repair pathways while defects in these pathways confer susceptibility to treatment [106].

Ring finger protein 8 (RNF8) is a RING finger E3 ligase involved in NHEJ repair. RNF8 regulates the level of Ku80 at DSB sites, making it an essential protein in the efficiency of NHEJ repair [107]. In fact, this protein has been shown to promote metastasis in breast cancer and its overexpression in lung cancer enhances the epithelial-mesenchymal transition, correlating with an increased risk of metastasis [108]. RNF8 overexpression has also been found in cisplatin-resistant endometrial cancer (EC). A recent study showed that RNF8 knockout significantly reduces NHEJ efficiency in chemoresistant EC. These results were supported in a cisplatin-resistant EC mouse model demonstrating promotion of cisplatin response by RNF8 deficiency [107]. Although in vitro and in vivo evidence highlight RNF8 as a potential treatment target in reducing resistance, an RNF8 inhibitor has yet to be developed and will require further investigation [107].

Targeting components of HR provides another mechanism through which cisplatin treatment can be improved. A leiomyosarcoma genomic and transcriptomic study found that leiomyosarcoma cells enriched with mutational signatures for defective HR demonstrated sensitivity not only to cisplatin, but also to a class of drug call poly (ADP-ribose) polymerase (PARP) inhibitor [109]. PARP is a key enzyme in the repair of single stranded breaks (SSBs) and promotion of HR when DSBs are present. The role of PARP inhibitors (PARPi) in cisplatin treatment is to inhibit PARP-dependent repair mechanisms, leading to the accumulation of cisplatin-related DNA damage and apoptosis. PARP inhibition is cytotoxic to tumor cells that are deficient in HR function [110]. Olaparib is one PARPi that is approved for the maintenance of HR-deficient high-grade serous ovarian cancer (HGSOC). However, PARPi treatment fails to control HGSOC proficient in HR. About 20% of HR-proficient HGSOC has overexpression of coactivator-associated arginine methyltransferase 1 (CARM1), an arginine methyltransferase oncogene involved in epigenetic regulation and gene transcription [111,112]. CARM1 silences the expression of MAD2L2, a subunit of the shieldin complex that is critical in determination between HR and NHEJ repair. A study found that the use of enhancer of zeste homolog 2 (EZH2) inhibitors will increase the expression of MAD2L2 in high-CARM1 HR-proficient HGSOC, sensitizing tumor cells to PARPi treatment [113]. Similarly, another study showed that small molecule inhibition of bromodomain containing 4 (BRD4), a protein involved in the facilitation of oncogene expression, in many HR-proficient tumor cell lines sensitizes cells to PARPi treatment [114]. Moreover, PARPi have been shown to act synergistically with cisplatin and significantly reduce the concentration needed to induce cytotoxic effects in various cancer cell lines [115]. Potential cisplatin sensitization by PARPi is supported by clinical data as well. Niraparib is one PARPi used alongside cisplatin that improved PFS in patients with advanced ovarian cancer. Approximately 25% of HGSOCs are defective in HR repair mechanisms, often due to BRCA1/2 germline and somatic mutations that make treatment with PARPi and DNA-damaging agents a suitable option [116,117]. Olaparib, niraparib, and veliparib are involved clinical trials to assess their enhancement of cisplatin treatment [118] (Table 1).

ATM, a protein involved in the enhancement of HR following DSB damage, is another potential target of therapeutics [119]. Studies have shown that low expression of ATM enhances sensitivity to not only ATM inhibitors, but also PARPi treatment [120,121,122,123]. Several molecules have been identified as ATM inhibitors. Two of these, AZD0156 and AZD1390 (ClinicalTrials.gov Identifier: NCT02588105, ClinicalTrials.gov Identifier: NCT03423628), are involved in clinical trials to assess their effectiveness in monotherapy and combination therapy with radiation and other chemotherapeutics. While none of these trials investigate ATM inhibitor use in cisplatin-treated cancers, the results might support future expansion to cisplatin.

Spironolactone (SP), a mineralocorticoid and androgen receptor antagonist commonly used in the treatment of hormonal acne, heart failure, and hypertension, is a small molecule inhibitor that enhances the degradation of XPB [124,125,126,127]. In vitro studies demonstrated SP inhibited NER and sensitized human ovarian and colon cancer cells to cisplatin and oxaliplatin [124]. On the other hand, SP has been shown to decrease the removal of UV photoproducts in skin cancers, raising concern that it could increase the risk of mutagenesis and carcinogenesis even at low concentrations [127]. Therefore, further investigation is needed to determine whether SP can be used to target chemoresistant cancer cells without increasing the risk of developing additional cancers.

Other NER targets of interest include XPA and XPF. XPA is a scaffold protein necessary for the assembly of the pre-incision complex on damaged DNA [55]. Low expression of XPA in metastatic testicular cancer is associated with better prognosis and superior OS compared to those with higher XPA expression [58]. Using computer-aided screening of XPA protein structure and a library of small molecule inhibitors, 63 molecules were identified to target the XPA DNA-binding domain [128]. Among these, X80 inhibits the binding of XPA to single-stranded and double-stranded DNA cisplatin lesions [128]. XPA also plays an important role in the association between ERCC1 and XPF by interacting with ERCC1 and recruiting XPF. UCN-01 is a cell cycle checkpoint inhibitor that inhibits the interaction of XPA with ERCC1 [129]. Two molecules have also been found to inhibit the XPF repair endonuclease. These two molecules do not inhibit other endonucleases or the ability of ERCC1-XPF to bind to DNA, indicating high specificity for XPF endonuclease activity [130]. A study using a lung cancer xenograft mouse model showed that combination treatment of cisplatin and XPF inhibitor NSC16168 significantly inhibited tumor growth compared to monotherapy with cisplatin or inhibitor [130]. While these small molecule inhibitors show promising results in multiple studies, they have not been studied clinically. Therefore, further investigation into NER inhibitors is necessary to truly understand their potential in improving cisplatin treatment.

Proteins within the BER pathway have also been implicated in cisplatin resistance. APE1 is a protein commonly elevated in NSCLC and is associated with poor PFS following platinum-agent chemotherapy. Preclinical data identifies an APE1 inhibitor, no. 0449-0145, which induces DNA damage and apoptosis in two NSCLC cell lines. Moreover, the inhibitor overcame cisplatin resistance in the cell lines. In vivo studies demonstrated inhibition of NSCLC progression in mice. These promising results following APE1 inhibition prompted further investigations into various small molecule inhibitors of the protein [131]. Gossypol is an inhibitor of APE1 involved in a phase III trial (ClinicalTrials.gov Identifier: NCT01977209) to assess its ability to improve the sensitivity of cisplatin-based chemotherapy in NSCLC patients with high APE1 expression (Table 1). The molecule is a Bcl-2 homology 3 (BH3)-mimetic agent shown to directly interact with the Bcl-2 homology (BH) domains of APE1, inhibiting the endonuclease’s repair activity [132]. A study consisting of sixty-two advanced NSCLC patients with no previous history of platinum-based chemotherapy showed that patients treated with gossypol along with the assigned docetaxel and cisplatin treatment experienced greater PFS and OS than those in the control group receiving placebo along with the same docetaxel and cisplatin regimen. Though there were no significant differences in PFS, OS, and adverse events between treatment and control groups, the addition of gossypol was well tolerated, and the results encourage larger studies to investigate the drug’s therapeutic potential [133].

Flap endonuclease 1 (FEN1), a member of the XPG/RAD2 endonuclease family, is another protein involved in BER whose overexpression in cancer is associated with an aggressive phenotype and platinum-agent resistance. A pre-clinical study showed that the depletion of FEN1 sensitizes previously resistant epithelial ovarian cancer cells and identified several small molecules that could be developed as FEN1 inhibitors [134].

With the growing recognition that DDR manipulation provides a successful route for overcoming cisplatin resistance, many agents have been developed as adjuvants for cisplatin. Cell cycle checkpoint signaling is vital for the coordination of DDR and cell cycle. CHK1 has proven to be a clinically useful target that works in concert with ATR to ensure G_2_/M cell cycle arrest during DNA repair [135]. It also stabilizes replication forks and participates in nuclear translocation and interaction with HR proteins BRCA2 and RAD51 [135]. Prexasertib is a CHK1 inhibitor that can be used to abrogate DNA damage and has shown promise in the treatment of cisplatin-resistant cancers. However, tumors can develop resistance to this drug, and investigation of the underlying mechanism is underway [136]. Prexasertib is currently involved in a phase I clinical trial (ClinicalTrials.gov Identifier: NCT02555644) to assess its effectiveness in combination with cisplatin to treat locally advanced head and neck cancer (Table 1).

Inhibition of CDK4/6 has also been indicated in the treatment of cisplatin-resistant cancers. The CDK4/6 pathway enhances cell cycle progression in a variety of cancers, such as liposarcoma, rhabdomyosarcoma, NSCLC, glioblastoma, esophageal cancer, melanoma, and breast cancer [137]. CDK4/6 inhibition results in the repression of HR proteins, which might serve as a mechanism in cisplatin sensitization [138]. Palbociclib and ribociclib are CDK4/6 inhibitors that have been evaluated in clinical trials [139]. Currently, two trials are investigating the use of palbociclib in combination with cisplatin (Table 1). The first is a phase I study (ClinicalTrials.gov Identifier: NCT02897375) investigating toxicity and optimal dosing of palbociclib with cisplatin or carboplatin in patients with metastatic solid tumors (Table 1). The second studies (ClinicalTrials.gov Identifier: NCT03389477) patients with HPV-negative HNSCC treated with neoadjuvant palbociclib, followed by chemoradiation and then adjuvant palbociclib (Table 1). While it is not currently being investigated in combination with cisplatin, a phase I trial (ClinicalTrials.gov Identifier: NCT03056833) is investigating the combination of ribociclib with carboplatin in platinum-sensitive ovarian cancers. One study evaluated the use of palbociclib and ribociclib in the treatment of cisplatin resistant- and sensitive-germ cell tumors (GCTs). Both agents decreased tumor viability and promoted cell cycle arrest and apoptosis [140]. The high sensitivity of these tumors to CDK4/6 inhibitors indicates their potential in increasing the therapeutic index of cisplatin.

## 6. Conclusions

Despite remarkable advancements in cancer treatment, the challenges of chemotherapy resistance and toxicity remain significant and continue to hinder patient survival and quality of life. Improved understanding of the DNA repair pathways involved in cisplatin sensitivity, resistance, and toxicity could help maximize the therapeutic index of cisplatin and allow for better cancer control with decreased toxicity. Incorporation of preclinical data and patient-focused predictive markers could allow for the expansion of “precision oncology” and help guide treatment decisions based on a genomic pattern as opposed to single markers. The development of drugs that disrupt DNA repair pathways has significantly improved the efficacy of cisplatin in resistant cases, and further investigation of new small molecule inhibitors might expand on this progress. Combining better patient selection with more effective treatments could overcome some of the challenges faced by cancer patients today and impact future outcomes.

## Figures and Tables

**Table 1:** Clinical trials studying combination chemotherapy of inhibitors with cisplatin.

Target	Inhibitor	CT Identifier	Other Regimen	Phase	Enrollment	Recruitment Status
CHK1	Prexasertib	NCT02555644	Cetuximab, Radiation	I	70	Completed
		NCT02124148	Cetuximab, G-CSF, Pemetrexed, Fluorouracil, LY3023414, Leucovorin	I	167	Completed
CDK4/6	Palbociclib	NCT02897375	Carboplatin	I	90	Recruiting
		NCT03389477	Cetuximab, radiation	II	29	Recruiting
PARP	Olaparib	NCT02308072	Radiation	I	70	Active, not recruiting
		NCT02882308	Durvalumab	II	41	Completed
		NCT01562210	Radiation	I	28	Completed
		NCT01296763	Irinotecan, Mitomycin-C	I	18	Completed
		NCT00782574	-	I	56	Active, not recruiting
		NCT00678132	Gemcitabine	I	23	Completed
		NCT02533765	-	II	18	Active, not recruiting
	Niraparib	NCT03983226	Cisplatin/gemcitabine, Carboplatin/taxane, Carboplatin/gemcitabine, Liposome doxorubicin/carboplatin	II	96	Recruiting
	Veliparib	NCT01711541	Carboplatin, Fluorouracil, Hydroxyurea, Paclitaxel, Radiation	I, II	24	Active, not recruiting
		NCT02723864	VX-970	I	53	Active, not recruiting
		NCT02595905	-	II	333	Active, not recruiting
		NCT01104259	Vinorelbine tartrate	I	50	Completed
		NCT01585805	Gemcitabine, Gemcitabine hydrochloride	I	107	Active, not recruiting
		NCT01281852	Paclitaxel	I	37	Completed
		NCT01642251	Etoposide	I, II	156	Completed
		NCT01711541	Carboplatin, Fluorouracil, Hydroxyurea, Paclitaxel, Radiation	I, II	24	Active, not recruiting
APE1	Gossypol	NCT01977209	-	III	204	Unknown
